# Passive sensing of gait and medication-related fluctuations in Parkinson’s disease

**DOI:** 10.1186/s12984-026-02068-6

**Published:** 2026-06-23

**Authors:** Juyoung Jenna Yun, Charalambos Hadjipanayi, Arya Jahangiri, Alan Bannon, Timothy G. Constandinou, Shlomi Haar

**Affiliations:** 1 Department of Brain Sciences, Imperial College London, London, UK; 2 Care Research and Technology Centre, UK Dementia Research Institute, London, UK; 3 Department of Electrical and Electronic Engineering, Imperial College London, London, UK; 4 Department of Bioengineering, Imperial College London, London, UK; 5 School of Psychology, University of Surrey, Guildford, UK

## Abstract

**Background:**

Gait impairment is a hallmark symptom of Parkinson’s Disease (PD). Traditional clinical assessments cannot capture real-world motor fluctuations, as they are sparsely performed. We validated the use of nearables, passive sensing technologies, including Kinect RGB-D cameras and ultra-wideband (UWB) radar, for continuous, objective assessment of gait fluctuations in PD within a home-like setting.

**Methods:**

Fifteen PD patients with mild symptoms and fourteen age- and sex-matched healthy controls (HC) performed 4-metre walking tasks in a living lab facility. Patients repeated the task during “ON” and “OFF” states of their daily medication cycle. Gait features, including stride length, stride time, and gait speed, were extracted from Kinect, radar, and a ground-truth smart floor. Data were analysed to assess inter-sensor agreements and group-level differences.

**Results:**

Stride time demonstrated the highest agreement between devices (*r* = 0.903), while stride length was weaker (*r* = 0.779). Nevertheless, stride length from both Kinect and radar distinguished PD OFF from HC (camera *q* = 0.020; radar *q* = 0.005), and radar additionally differentiated ON from OFF (*q* = 0.020). Neither device differentiated PD ON from HC, indicating medication reduced observable gait differences.

**Conclusions:**

Although some spatial metrics show device discrepancies, both systems demonstrate sensitivity to gait patterns and medication-dependent changes, supporting their use for longitudinal, real-world monitoring of motor symptoms.

## Introduction

Gait disturbances are some of the most common and disabling motor symptoms in Parkinson’s Disease (PD), with freezing of gait affecting up to 80% of individuals with advanced disease [1, 2]. Beyond their high prevalence, these gait impairments carry profound clinical implications; they are a primary driver of falls, leading to increased hospitalisation rates and severely reduced quality of life [3, 4]. Furthermore, quantitative gait decline is increasingly recognised as a critical digital biomarker for overall disease progression [5, 6]. PD motor symptoms are highly multidimensional, varying considerably across individuals and fluctuating throughout the day in response to medications such as levodopa [7]. Currently, the gold standard for assessing disease severity and progression is the Unified Parkinson’s Disease Rating Scale (UPDRS). However, the motor examination part of the UPDRS (known as part III) has several well-recognised limitations, including intra- and inter-rater variability and structured tasks that may not reflect natural movement [8–10]. Moreover, the infrequent nature of this measurement during clinical visits limits the sensitivity to the full range of motor fluctuations occurring throughout the day [9].

Marker-based motion capture and instrumented walkways provide a gold standard for quantitative gait analysis [11–13]. Yet, those are expensive, unscalable setups. In contrast, wearable sensors have emerged as a cost-effective tool for quantifying gait and have become widely used in both clinical and home settings, providing accessible alternatives to traditional laboratory equipment [14–17].

The most common wearables are smartwatches, due to their familiarity and social acceptability, promoting higher compliance for daily use [18, 19]. Smartwatches can estimate gait parameters using their inertial measurement unit (IMU) [20, 21]. However, their accuracy can be strongly altered by arm swing patterns in PD patients or reduced arm swing in older adults [20, 22, 23]. Lumbar sensors offer better precision for step detection and temporal metrics and have shown potential in differentiating PD subtypes through gait biomarkers [24, 25]. However, these wearables are sensitive to accurate placement and consistent orientation on the lumbar spine, where small deviations can distort gait asymmetry and degrade data quality [26, 27]. Fundamentally, while wearable systems are highly capable, they remain insufficient for long-term, continuous home monitoring due to their inherent nature to rely on strict patient adherence, precise daily sensor placement by the user, and constant battery maintenance.

Passive motion tracking technologies are uniquely suited for free-living applications, as they provide uninterrupted and long-term insights into symptom progression without requiring charging, user interaction, or compliance, allowing a truly “install and forget” capability [28, 29]. RGB-depth cameras like the Microsoft Kinect yield spatiotemporal gait measurements that are comparable to gold-standard marker-based motion capture under controlled laboratory conditions [30–33]. These systems avoid the need for reflective markers and specialised laboratory setups or dedicated floor surfaces, and thus are practical for home deployments. Despite these benefits, their performance can be influenced by environmental factors such as lighting conditions and occlusions, posing challenges for protocol standardisation and measurement consistency [34–36].

Radar systems offer complementary advantages, including enhanced privacy and the ability to detect subtle movements even through clothing, with high temporal resolution to capture gait dynamics that can identify gait disorders or reveal early signs of neurological conditions such as PD [37–40]. Our prior work demonstrated that low-power and cost-effective ultra-wideband (UWB) radar can accurately quantify spatiotemporal gait parameters in healthy participants during both normal and asymmetric walking, yet each unit inherently provides a one-dimensional field of view, limiting the accuracy of spatial measurements, unless multiple sensors are combined [40, 41]. Despite these technological validations in healthy cohorts, the sensitivity of these passive sensors to medication-induced motor fluctuations in clinical PD populations has not been established.

Therefore, the primary objective of this study was to validate the feasibility and sensitivity of passive nearable sensors (Kinect cameras and UWB radars) for objective gait assessment in individuals with PD. Specifically, we aimed to: (1) determine the absolute agreement of these devices against reference measures, (2) evaluate their capacity to differentiate people with mild PD from healthy controls, and (3) assess their sensitivity to detect natural within-day motor fluctuations (ON vs. OFF medication states) in a fully functioning living environment. Importantly, our approach captures natural fluctuations observed in PD patients across the day without artificially inducing extreme medication states, providing a foundation for continuous, passive motion tracking in the home.

## Results

A total of 15 individuals with PD and 14 age- and sex-matched HC participants took part in the study (age: *p* = 0.346; sex: *p* = 0.836) (Table 1). PD symptoms severity fluctuated across the ON and OFF states of the participants’ medication cycle (Table 2). A significant difference in the UPDRS motor examination score between states (*p* = 0.020) reflects expected dopaminergic improvements in motor symptoms during ON medication. Gait item and symmetry indices derived from the UPDRS were also reported, with no significant differences between states.

**Table 1:** Participants’ demographics and clinical characteristics

	HC (*n* = 14)	PD (*n* = 15)	*P*-value
Age	70.8	68.0	0.346
Gender (male/total)	10/14	10/15	0.836
Handedness (right/left/both)	10/3/1	13/2/0	0.637
LEDD (mg) (min-max)	-	356 (37.5–850)	-

Group comparison between healthy controls (HC) and people with Parkinson’s disease (PD). Values represent group means. No significant differences were observed in age, gender distribution, or handedness between groups. Levodopa equivalent daily dose (LEDD) is shown for the PD group.

**Table 2:** Participants’ clinical characteristics

	PD ON (*n* = 15)	PD OFF (*n* = 15)	*P*-value	Magnitude of Change	Effect Size (Cohen’s d)
UPDRS III – Gait (mean ± SD)	0.53 ± 0.64	1.07 ± 0.80	0.055	0.53	1.10
UPDRS III – Total (mean ± SD)	12.7 ± 7.12	20.5 ± 10.5	**0.020**	7.87	0.72
Symmetry Index (R-L) (mean ± SD)	37.1 ± 36.2	54.5 ± 36.2	0.198	17.43	0.35

Comparison of PD participants’ UPDRS subscores and gait symmetry index between ON and OFF medication conditions.

### Study procedures

**Fig. 1:**
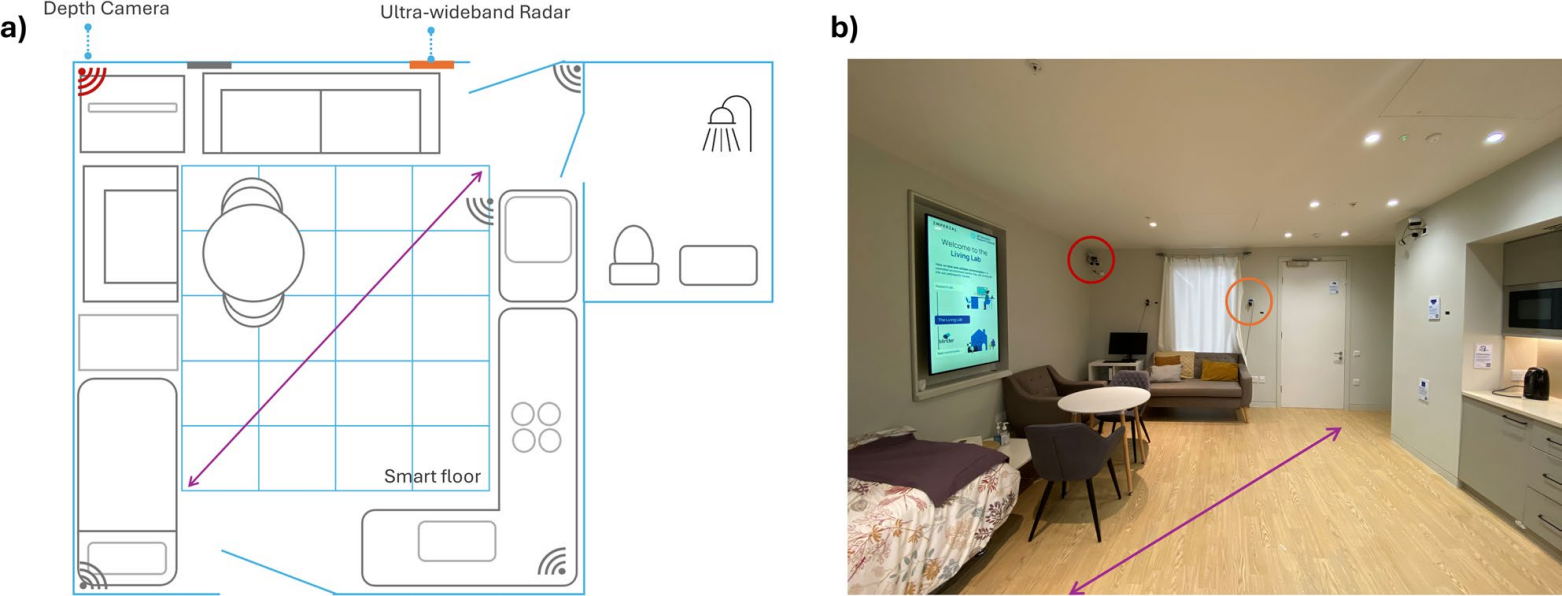
Environmental sensor layout in the Living Lab. **a** Schematic of the home-like experimental environment equipped with multimodal sensors. Ultra-wideband radars (rectangle icons – the one in orange was used here) and depth cameras (wave-emission icon – the one in red was used here) were installed around the room. The 4-metre walking pass is marked with a purple arrow. The smart floor system (blue grid) embedded beneath the floor tiles recorded participants’ location while walking. **b** Photograph of the Living Lab from the same perspective as panel (**a**). The radar is highlighted in an orange circle, whereas the camera is in a red circle. The walking trajectory is indicated again with a purple arrow

The study was conducted in the Living Lab at Imperial College London [42], a facility designed to replicate a fully functioning home environment with standard domestic furnishings and appliances (Fig. 1). For PD participants, each session was scheduled so that patients took their regular medication dose upon arrival, after which preliminary paperwork was completed while waiting for the medication to take effect. Motion data for the ON medication state were collected approximately one hour after medication intake. To capture the “wearing-off” (OFF) state, corresponding motion data were collected immediately before the patients’ next scheduled medication dose, representing the period of maximal symptom re-emergence within their daily cycle. In both ON and OFF states, motor symptom severity was assessed using the UPDRS motor examination (known as part III). In line with the UPDRS gait item, participants performed a straight walk approximating the 10-metre standard. For feasibility within the Living Lab, a 4 m path was used, allowing consistent observation of a minimum of four gait cycles per trial. HC participants performed the walking task once and without a full UPDRS motor examination.

During the assessment, three sensing modalities were operated simultaneously, including Microsoft Azure Kinect DK, Novelda XeThru UWB radar, and SensFloor room terminal systems. One Kinect camera and one UWB radar, selected based on their optimised placement relative to the walking path, were used in this study (positioning highlighted in Fig. 1). SensFloor data were used as the reference measure for walking speed alignment.

### Comparison with the floor reference

**Fig. 2:**
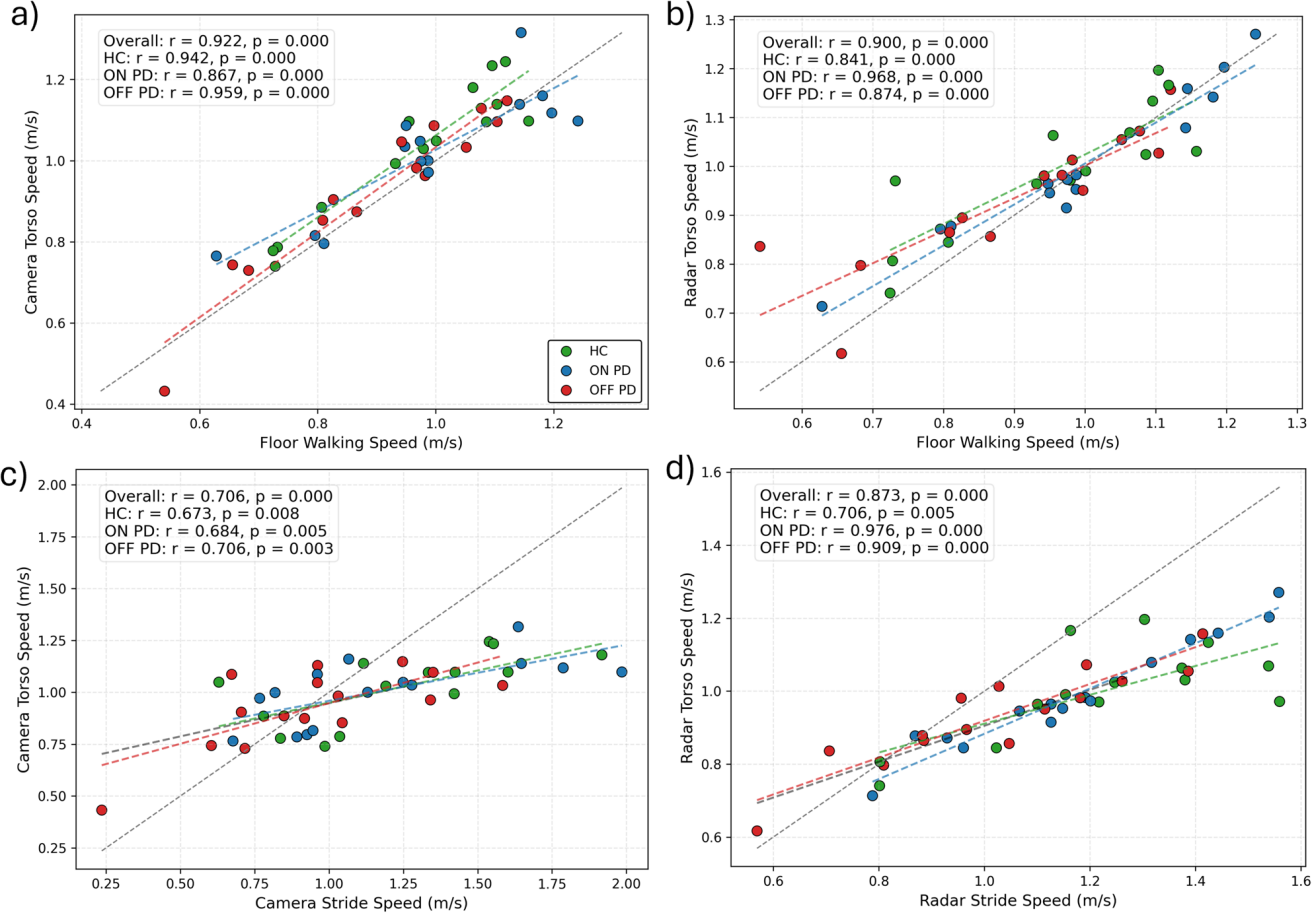
Measurements of walking speed across devices. **a**, **b** Correlations between smart floor walking speed and camera (**a**) or radar (**b**) derived torso speed for healthy controls (HC, green), and Parkinson’s participants (PD) in medication ON (blue), and OFF (red) states. Dashed lines denote linear fits, and the identity line is dotted black. Correlation coefficients (r) are reported for each group and overall. Correlation coefficients (r) are reported for each group and overall. **c–d** Correlations between stride- and torso-level walking speeds derived from the same modality – camera (**c**) or radar (**d**). Legends are the same as in (**a**)

Torso speed derived from camera and radar data was compared with floor-measured walking speed (Fig. 2a and b). Both modalities showed strong correlations with the floor measurements, with overall correlation coefficients exceeding 0.90 (*p* < 0.0001 for all states). The camera data yielded an overall RMSE of 0.05 m/s (5.25% of the mean) and *r* = 0.922 (HC: *r* = 0.942; ON PD: *r* = 0.867; OFF PD: *r* = 0.959) while radar showed a comparable performance with an overall RMSE of 0.081 m/s (8.45% of the mean) and *r* = 0.900 (HC: *r* = 0.841; ON PD: *r* = 0.968; OFF PD: *r* = 0.874).

### Agreement within each device

Within-device comparisons were conducted to examine how each sensing modality captures differences between torso speed and foot-derived stride speed (Fig. 2c and d). Camera-derived measures showed an RMSE of 0.104 m/s (10.5% of the mean) and overall correlation of *r* = 0.797, *p* < 0.0001 (HC: *r* = 0.779, *p* < 0.001; ON PD: *r* = 0.766, *p* < 0.001; OFF PD: *r* = 0.833, *p* < 0.0001), while radar demonstrated even better association with an overall RMSE of 0.067 m/s (6.88% of the mean) and *r* = 0.873, *p* < 0.0001 (HC: *r* = 0.706, *p* < 0.01; ON PD: *r* = 0.976, *p* < 0.0001; OFF PD: *r* = 0.909, *p* < 0.0001). The camera data showed a slightly greater variability around the regression line at higher stride speeds compared to radar. The slope of the regression lines deviated from the identity line in both modalities, indicating systematically higher stride speeds compared to torso speeds.

### Agreement between devices

**Fig. 3:**
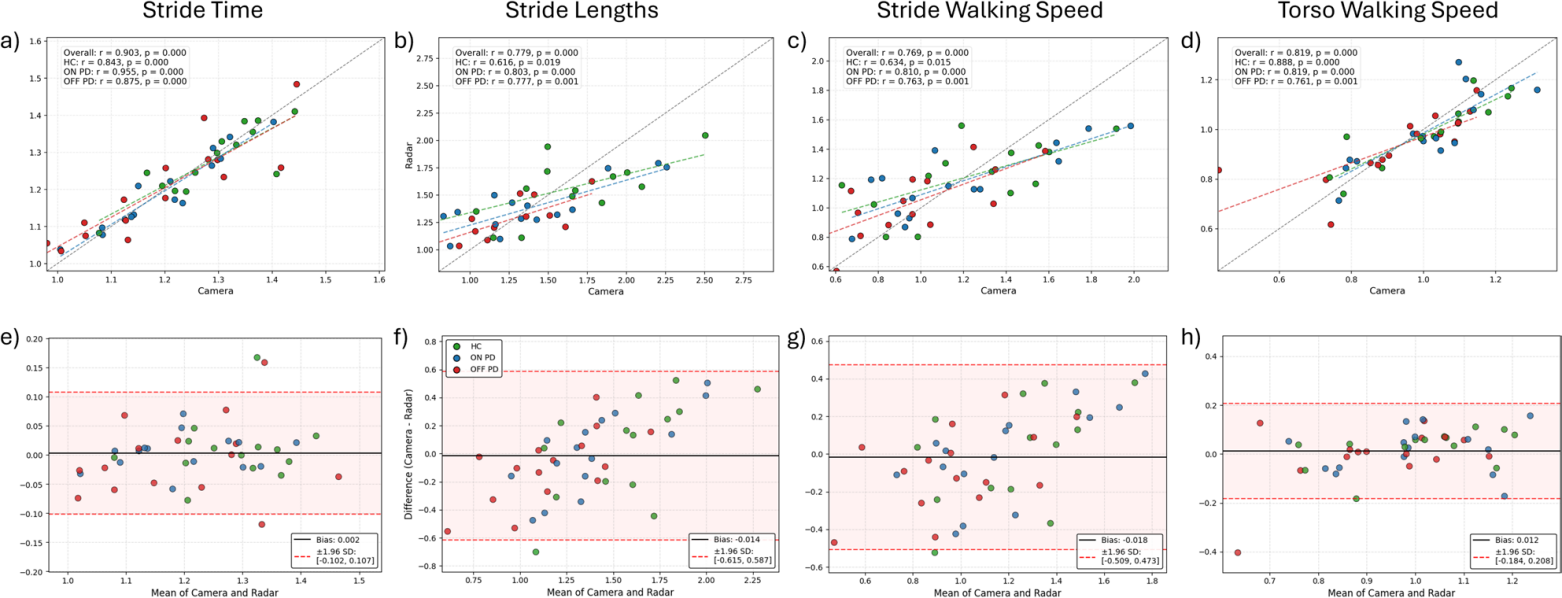
Gait metrics across sensing modalities. Top row shows correlations between camera- and radar-derived metrics: **a** stride time, **b** stride length, **c** stride speed, and **d** torso speed. Legends are the same as in Fig. 2. The bottom row presents the agreement between camera- and radar-derived metrics in Bland-Altman plots

Figure 3 illustrates four key gait-related features, including stride length, stride time, stride speed, and torso speed, compared between camera and radar. Among these, stride time (Fig. 3a) showed the strongest inter-device association with RMSE = 0.048s (3.96% of the mean) and *r* = 0.903 (*p* < 0.0001), demonstrating robust temporal agreement between devices. Stride length (Fig. 3b) showed a slightly weaker agreement between devices, with RMSE = 0.169 m (12.2% of the mean) and *r* = 0.779 (*p* < 0.0001), where camera measurements tended to overestimate distances relative to radar. Stride speed (Fig. 3c), which is a function of stride length and time, displayed a very similar trend with RMSE = 0.156 m/s (13.68% of mean) and *r* = 0.769 (*p* < 0.0001). The torso speed (Fig. 3d) demonstrated slightly strong agreement between devices with RMSE = 0.079 m/s (8.10% of the mean) and *r* = 0.819 (*p* < 0.0001).

To assess absolute agreement between the calibrated camera system and the reference radar, 44 paired walking trials were evaluated using Bland-Altman limits of agreement and ICC(A,1) (Fig. 3e-h). Temporal metrics showed excellent inter-device agreement, with stride time yielding an ICC of 0.90 and a negligible systematic bias of 0.00 s (95% Limits of Agreement: [-0.10, 0.11]). Following upstream spatial calibration, camera-derived stride length and walking speed demonstrated good agreement with the radar (ICC = 0.68 and 0.870, respectively) and successfully minimised systematic bias to -0.01 m and − 0.02 m/s, respectively. Torso walking speed also demonstrated good agreement between devices (ICC = 0.80, bias = 0.01 m/s).

### Group and state differences

**Fig. 4:**
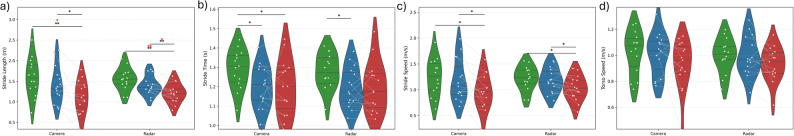
Gait metrics across groups. Violin plots comparing the metrices across the groups, healthy controls (green), and Parkinson’s participants in medication ON (blue), and OFF (red) states. Each plot shows camera (left) and radar (right) derived metrics: **a** stride length, **b** stride time, **c** stride speed, and **d** torso speed. Black asterisks mark uncorrected statistically significant differences (*p* < 0.05), and red asterisks mark FDR-corrected significant differences (q < 0.05). For each asterisk, p/q < 0.05 (*), < 0.01 (**),<0.001 (***)

For stride length (Fig. 4a), the radar successfully distinguished differences between HC and PD OFF participants (*q* = 0.005) and between PD ON and OFF states (*q* = 0.024). Similarly, the camera showed a weaker but still significant difference between HC and PD OFF participants (*q* = 0.025) but could not significantly discriminate between ON and OFF states after correction for multiple comparisons (*p* = 0.018, *q* = 0.051). Neither device differentiated HC from PD ON participants. For stride time (Fig. 4b), group-level differences were modest, with camera data showing nominal differences between HC and PD ON (*p* = 0.032, *q* = 0.128) and between HC and PD OFF (*p* = 0.049, *q* = 0.066), and radar showing a difference between HC and PD ON (*p* = 0.031, *q* = 0.124); however, none reached significance after false discovery rate (FDR) correction. For stride speed (Fig. 4c), radar showed nominal differences between HC and PD OFF (*p* = 0.037, *q* = 0.074) and between PD ON and OFF (*p* = 0.027, *q* = 0.053) and camera showed similar nominal trends for HC and PD OFF (*p* = 0.035, *q* = 0.066)) and PD ON and PD OFF (*p* = 0.026, *q* = 0.051), though again these did not survive the FDR correction. Finally, for torso speed (Fig. 4d), there were no significant group-dependent differences.

### Symmetry index comparison

**Fig. 5:**
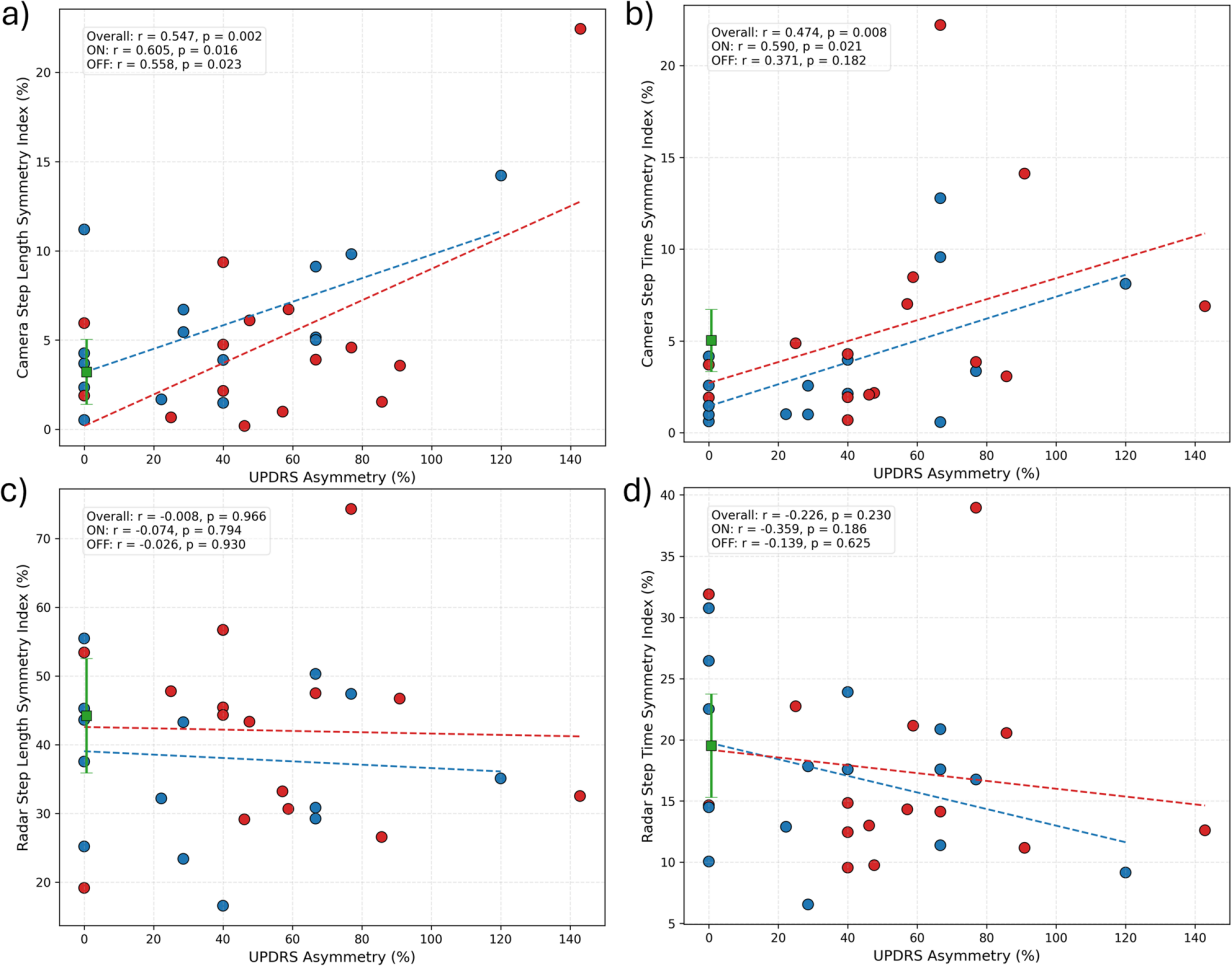
Gait symmetry correlations between clinical and sensor-derived measures. **a** Correlations between UPDRS III gait symmetry scores and camera-based step-length symmetry (left) and step-time symmetry (right). **b** Equivalent correlations for radar-based indices. Each point represents a Parkinson’s participant in the ON (blue) or OFF (red) state. Sensor-derived gait symmetry indices for the healthy control group are presented as green error bars at the bottom of each panel

The relationship between the gait symmetry index and UPDRS motor symmetry index scores was assessed for both camera (Fig. 5a and b) and radar (Fig. 5c and d) data. Camera-derived step length asymmetry showed a moderate positive correlation with UPDRS asymmetry (*r* = 0.547, *p* = 0.002), with significant associations in both medication states (PD ON: *r* = 0.605, *p* = 0.016; PD OFF: *r* = 0.558, *p* = 0.023). Yet, this was highly variable (overall RMSE = 3.85 against a mean of 5.30%, representing 72.6% error). Step time asymmetry from the camera also showed a positive association (*r* = 0.474, *p* = 0.008), which was significant in the ON state (*r* = 0.590, *p* = 0.021) but not in the OFF state (*r* = 0.371, *p* = 0.182), yet again, highly variable (overall RMSE = 4.17 against a mean of 4.74%, representing 88.1% error). The HC step metrics from the camera are shown as 95% reference intervals: [1.38, 5.03] for length asymmetry and [3.33, 6.71] for step time asymmetry, and overlap with the lower end of PD asymmetry. In contrast, radar-derived metrics for step length and step time asymmetry demonstrated no significant relationship with UPDRS asymmetry. Step length symmetry from radar was uncorrelated with clinical asymmetry (*r* = − 0.008, *p* = 0.966; PD ON: *r* = − 0.074, *p* = 0.794; PD OFF: *r* = − 0.026, *p* = 0.930), and step time symmetry exhibited a negligible negative association (*r* = − 0.226, *p* = 0.230; PD ON: *r* = − 0.359, *p* = 0.186; PD OFF: *r* = − 0.139, *p* = 0.625). The 95% reference intervals for HC radar data were [35.86, 52.52] for step length asymmetry and [15.30, 23.74] for step time asymmetry, and overlapped with the middle to high end of PD asymmetry, indicating limitations in extracting precise asymmetry measures from a single radar configuration.

## Discussion

This study demonstrates the inter-device agreement and demonstrates the usability of nearables, passive motion-sensing devices, for tracking gait patterns in PD. We demonstrate the sensitivity to differentiate people with mild PD in their OFF state from healthy older adults and within-patient sensitivity to changes in specific gait parameters over their daily medication cycle. Prior gait studies in PD have relied on structured laboratory protocols or wearable sensors that require active participation. Our findings demonstrate that passive, non-contact sensing is sensitive to disease-related and medication-related gait alterations in an ecologically valid environment, without burdening the participant. Additionally, the study reports step-level asymmetric measures, with a notable association observed in camera-derived metrics. Overall, these findings exhibit the feasibility of using passive sensors to capture clinically meaningful motor changes in PD.

While there is an increase in the use of wearable devices in Parkinson’s patients, including recent guidelines from the UK’s National Institute for Health and Care Excellence (NICE) [43], those devices can burden patients. Recent research addresses the practical challenges of deploying wearable sensor systems for long-term, continuous monitoring in real-world environments [24]. Although feasible for monitoring mobility and medication adherence, technical and usability issues reduced data completeness and satisfaction [44]. Older participants reported greater difficulty using the system. Similar trends have been reported in other sensor-based studies, where robustness, validation in unsupervised settings, and accessibility remain major barriers [45–47].

Passive sensing technologies show promising potential for addressing these limitations. Our study demonstrates consistent within-device and between-device performance despite inherent modality-specific variability. When exploring within-device dynamics, camera data showed higher variability around the regression line at higher stride speeds, whereas radar measurements were more tightly clustered, indicating potentially higher consistency in velocity estimation. Furthermore, the slope of the regression lines deviating from the identity line in both modalities indicated systematically higher stride speeds compared to torso speeds. This reflects the expected difference between limb-driven forward thrust and centre of mass translation, highlighting the distinction between segmental limb motion and whole-body movement, as torso-based measures primarily capture postural displacement rather than full gait dynamics.

Moreover, both camera and radar demonstrated high sensitivity in discriminating PD OFF from HC based on stride length metrics. Stride length impairment is a well-established and significant marker of motor deficits in PD. Multiple studies reported stride length reductions with disease progression, while stride frequency tends to remain relatively stable or variably affected [1, 48, 49]. Dysfunction in basal ganglia–cortical circuits disrupts movement amplitude, as reflected in improvements in gait symptoms and parameters when these pathways are targeted with dopaminergic therapy [50–53]. In our results, radar exhibited the most robust sensitivity in detecting both group-level differences between PD OFF and HC, and within-participant differences between PD medication states. However, it should be noted that this statistical sensitivity was predominantly driven by stride length; other metrics, such as stride speed and torso speed, showed only nominal differences that did not consistently survive FDR correction.

Although mild gait asymmetry occurs in healthy individuals, temporal asymmetries increase in early PD, and step-level metrics provide a more reliable assessment of symmetry as they directly capture interlimb coordination [54–56]. Increased gait asymmetry is considered a key contributor to motor instability in PD and may amplify symptoms such as balance loss, especially when not considered during interventions like deep brain stimulation (DBS) [57, 58]. For instance, stimulation of the subthalamic nucleus (STN) has been reported to induce increased step time asymmetry and dyscoordination in some patients [59]. The symmetry index in this study quantified the magnitude of asymmetry, revealing a moderately strong correlation between camera-derived step length asymmetry and the UPDRS motor asymmetry index. This association suggests that the camera-based metrics captured clinically meaningful limb imbalance, consistent with established lateralised motor dysfunction in PD. However, it is important to note that the relative RMSE for camera-derived asymmetry appeared high (72–88%). This was due to the cohort’s exceptionally low baseline asymmetry (5.3%), where minor absolute tracking errors (3.8%) heavily skew relative percentages. Clinically, this indicates that while the camera captures broader correlational trends in limb imbalance, it currently lacks the precision required to track absolute bilateral micro-fluctuations in early-stage PD. Our radar asymmetry metric failed to capture it here. While this is likely due to the inherent limitation of capturing individual limbs with a single radar [41], other contributing factors such as signal noise, segmentation accuracy, and the limited sample size must also be acknowledged. Future work will integrate multiple sensors or receivers into a scalable system to enable limb-specific sensing, enhance asymmetry detection, and capture more gait features.

While providing within-patient evidence for device sensitivity to gait fluctuations, the statistical power might have been limited by the sample size, and the generalisability to advanced PD might be limited by the mild symptoms of our participants. Additionally, the participants in this cohort were predominantly male, which may limit the generalisability of these findings to the broader female PD population. Yet, the main limitation of this study is that while it was performed in a home-like environment, it was not in the home, and the walk was instructed. Future studies should replicate this finding in patients’ homes while they walk around the house without being instructed. In addition, further validation, including comparisons to common wearable systems, would strengthen the validity and reproducibility of the passive sensing approach. Finally, the short walking distance (4 m) constrains the assessment of continuous gait variability and turning dynamics, which should be addressed in future activities of daily living.

## Conclusion

By capturing disease- and medication-related changes passively, radar and camera systems could complement clinic-based UPDRS assessments to provide objective, longitudinal markers of motor symptoms and their fluctuations. While camera-derived metrics capture spatial asymmetry linked to UPDRS-rated motor lateralisation, radar offers enhanced sensitivity to dopaminergic state changes, particularly through stride length evaluation. Together, these passive sensing modalities provide a foundation for continuous, contact-free motor assessment, advancing the development of digital biomarkers for PD management in the home.

## Methods

### Participants and ethical information

We recruited 15 individuals with PD and 14 age-matched HC. The sample size was set based on a priori power calculation for the within-participant paired test sensitivity, expecting a high effect size due to symptom fluctuations, and aligning with typical cohort sizes for early-stage sensor validation studies. The effect size was calculated based on the UPDRS scores, under the hypothesis that the sensors are expected to demonstrate comparable or greater sensitivity. Assuming UPDRS difference of 10 ± 10 between the on and off states and 0.9 power target. For both PD and HC, exclusion criteria included any neurological disorders (other than PD for the patient group) or gait impairments (e.g., severe orthopaedic conditions, recent joint replacements) that could influence locomotor performance. Inclusion criteria for the PD cohort required patients to be in the early-to-mild stages of the disease (defined as an UPDRS Part III motor score of ≤ 32, corresponding to mild motor impairment [60]), able to walk independently without walking aids, and on a stable dopaminergic medication regimen.

### Experimental setup and protocol

The study was conducted in a living laboratory environment designed to simulate a home-like setting while allowing for controlled sensor data collection. In the gait task presented here, participants were instructed to walk along a dedicated, predefined 4-metre straight path within the sensing area of the devices. Prior to each trial, a researcher provided standardised verbal instructions. Participants were specifically instructed to walk at their normal, comfortable, everyday pace (self-selected walking speed) rather than a fast-paced or artificially slow speed. They were told to look straight ahead and walk continuously past the marked end of the path to prevent deceleration artifacts within the capture volume. For the PD cohort, this exact protocol was conducted during both their ON and OFF medication states to capture clinical motor fluctuations.

### Camera data

The Azure Kinect DK captured 3D skeletal data from 32 joints at 30 Hz. Data were recorded as timestamped JSON files and processed in Python 3.12. The preprocessing pipeline included manual validation of walk trial timestamps, temporal segmentation, and participant identification to exclude any false detections. To suppress high-frequency noise, spatial trajectories were smoothed using a 4th -order zero-lag low-pass Butterworth filter with a 3 Hz cutoff frequency. Initial contact events were identified from alternating peaks and troughs in the filtered anterior-posterior distance between the left and right ankles, utilising minimum spacing and height constraints. These detected sequences were subsequently validated against specific time and length thresholds, and neighbour-difference outliers were removed. A minimum inclusion gate of 8 valid steps per trial was required. The three-dimensional joint positions of the pelvis and bilateral ankles were transformed into a walking-aligned coordinate frame based on the pelvis trajectory and floor orientation. Initial contact events were identified from alternating extrema in the filtered anterior–posterior distance between the left and right ankles, from which the step and stride gait parameters were derived.

During initial exploratory analyses, we identified a consistent overestimation of spatial gait parameters by the Kinect skeleton-tracking algorithm compared to the torso-based reflection centroid used by the reference radar. To resolve this, we utilised an upstream linear calibration. We derived calibration constants (slope and intercept) based on uncalibrated trials and automatically applied this mathematical transformation $$\:(Calibrated\_Metric\:=$$$$ slope\times\:Raw\_Metric\:+\:intercept)$$ to all Kinect spatial data before the final agreement (ICC/Bland-Altman) and medication-state statistical analyses. Furthermore, agreement was assessed using Intraclass Correlation Coefficients (ICC A,1; two-way random effects, absolute agreement) and Bland-Altman limits of agreement to quantify systematic bias.

### Radar data

Radar data were acquired from the sensor positioned closer to the walking path, whose sensing direction was more closely aligned with the walking trajectory and therefore provided higher accuracy when estimating movement along the path length [41]. Radar signals were sampled at 500 Hz with a distance range resolution of 5.1 cm. Signal acquisition, filtering, and post-processing followed the same pipeline as our previously published work [41], implemented in MATLAB (R2024a). This included suppression of reflections from stationary objects using adaptive exponential moving average filtering (coefficient constrained between 0.2 and 0.8) and second-order high-pass Butterworth filtering (cutoff velocity 0.05 m/s), and suppression of background noise using matched filtering. Following quadrature demodulation, range–Doppler representations were computed using short-time Fourier transforms (0.2-s Kaiser window, roll-off coefficient 10, 95% overlap), with contrast enhancement applied prior to percentile-based envelope extraction (2.5th–97.5th percentiles). From the processed data, relative distance (range) and velocity (Doppler) trajectories of the torso and feet were extracted. Because the walking path was not perfectly aligned with the sensor’s primary viewing direction, a geometric correction was applied to reflect movement along the walking direction. Initial contact events were identified as local minima in the feet velocity trajectories. The distance and timing information of the torso and initial contact events were then used to estimate the selected gait parameters.

### Floor data

SensFloor (Future-Shape GmbH) is a capacitive sensor system. It consists of a 3 mm thick textile underlay with built-in sensor electronics. SensFloor measures, through a flooring layer on top of it, changes in the electric capacitances on a triangular grid of sensor fields [61]. SensFloor “tracks” position at an approximately 32 cm*32 cm spatial resolution and a temporal resolution of 100ms. From these data, we derived speed (m/s) by calculating successive x and y coordinates for the same object during the 4 m walk. Given its relatively coarse spatial and temporal resolution and its inability to directly detect foot contacts, SensFloor cannot extract highly precise step-level kinematics. Consequently, it was used explicitly as a reference measure for macroscopic torso speed alignment, rather than as an absolute ground-truth for all gait dynamics.

### Trial alignment and synchronisation

The camera, radar, and floor systems inherently operate at different sampling rates. The primary validation framework of this study focused on aggregate, trial-level gait parameters (e.g., mean stride length, mean walking speed), continuous point-by-point temporal interpolation between the hardware streams was not required. Instead, data from each device was temporally segmented into matching walking trials. Gait events and features were extracted independently using each device’s specific preprocessing pipeline, and the resulting trial-averaged metrics were subsequently matched across devices for agreement analyses.

### Gait metrics

Step length was defined as the anterior–posterior distance (in metres) between initial contacts of opposite feet, whereas stride length is the distance covered by the same foot between two successive initial contacts. Individual step lengths are calculated as the distance between consecutive initial contact points, and stride length is computed as the sum of two consecutive step lengths. Stride length is reported as the mean of all valid values across the recording session. Step time is defined as the temporal interval (in seconds) between initial contacts of opposite feet, whereas stride time is the interval between two successive initial contacts of the same foot. Step times are calculated as the time interval between consecutive initial contact points, and stride time is derived as the sum of two consecutive step times. Mean stride time is then computed from all valid strides per participant.

Stride (or gait) speed is defined as the average linear velocity (in metres/second) of a complete stride, derived from detected gait events and reflecting the mean forward walking speed during a gait cycle. It is calculated as stride length divided by stride time, computed separately for each stride and then averaged across all strides in each session. Pelvis or torso speed is defined as the instantaneous linear velocity of the pelvis joint or torso during walking, computed directly from continuous joint trajectories as a kinematic measure of the body’s centre movement in the walking direction. It is derived from the time-differentiated displacement of the pelvis joint (Kinect) or torso (radar) in the walking direction, and mean pelvis or torso speed is reported across all valid gait segments for each participant. When SensFloor data are available, torso speed is estimated from the temporal sequence of pressure activations as the centre of pressure progresses forward across sensor tiles in the walking direction, allowing calculation of mean forward speed over the gait cycle.

Symmetry index (SI) is defined as the magnitude of gait symmetry between the two legs. This is computed using step length and step time, as these directly reflect inter-limb coordination and asymmetry between sides. Following preprocessing (which required a minimum of 8 valid steps and removed neighbour-difference outliers), the mean step values for the left and right sides were exported. SI was then derived from these mean lateralised primitives. Since radar cannot distinguish between which step originates from which leg (left/right or paretic/non-paretic), the absolute value was taken to make the metric direction-agnostic. Following the definition by Patterson et al. [62], this approach was also applied to camera and UPDRS data, thereby expressing only the magnitude of asymmetry as:

$$\:SI=\frac{\left|{Feature}_{leg1}-{Feature}_{leg2}\right|}{0.5\left({Feature}_{leg1}+{Feature}_{leg2}\right)}\times\:100\%$$

### Statistical analysis

Statistical analyses were performed using Python. The study employed a mixed design (between-subject factor: Group [PD vs. HC]; within-subject factor: Medication State [ON vs. OFF]). Descriptive statistics were calculated for movement data obtained from each device, and results for continuous variables are presented as mean ± standard deviation (SD). Prior to parametric analyses, data were tested for normality using the Shapiro–Wilk and Anderson–Darling tests, supplemented by visual inspection of Q-Q plots to ensure robustness given the sample size, and for homogeneity of variance using Levene’s and Bartlett’s tests.

Agreement between devices was assessed using Intraclass Correlation Coefficients (ICC A,1; two-way random effects, absolute agreement) and Bland-Altman limits of agreement to quantify systematic bias. While using the parametric Pearson’s r, robustness was validated through leave-one-out (LOO) resampling. This approach ensured the stability of the correlation by identifying potential outlier-driven influences. This was further reported with Root Mean Square Error (RMSE) of best fit alongside mean percentage.

To evaluate clinical differences, independent t-tests were used for between-group comparisons (PD OFF vs. HC), and paired t-tests were used for within-subject comparisons (PD ON vs. PD OFF). While linear mixed-effects models can accommodate mixed designs, we opted for planned pairwise comparisons to directly address our hypotheses regarding specific drug and disease effects, ensuring straightforward interpretation of the medication response. Statistical significance was defined as *p* < 0.05. For analyses involving multiple comparisons, q-values were calculated using FDR correction via the Benjamini-Hochberg procedure. To appropriately control the Type I error rate without over-penalising, this correction was applied separately within each specific comparison family across the four primary gait metrics.

## Data Availability

Data produced in the study are available upon reasonable request to the authors. The underlying code for this study will be made available to qualified researchers at reasonable request from the corresponding author.
